# A comparison of university student and community gamblers: Motivations, impulsivity, and gambling cognitions

**DOI:** 10.1556/JBA.3.2014.007

**Published:** 2014-02-03

**Authors:** Harvey H. C. Marmurek, Jessica Switzer, Joshua D’Alvise

**Affiliations:** ^1^Department of Psychology, University of Guelph, Guelph, Ontario, Canada; ^2^Now at the University of Calgary, Canada; ^3^Department of Marketing and Consumer Studies, University of Guelph, Guelph, Ontario, Canada

**Keywords:** motivation, impulsivity, gambling cognitions, gambler’s fallacy, problem gambling

## Abstract

*Background and aims:* The present study tested whether the associations among motivational, cognitive, and personality correlates of problem gambling severity differed across university student gamblers (*n* = 123) and gamblers in the general adult community (*n* = 113). *Methods:* The participants completed a survey that included standardized measures of gambling motivation, gambling related cognitions, and impulsivity. The survey also asked participants to report the forms of gambling in which they engaged to test whether gambling involvement (number of different forms of gambling) was related to problem gambling severity. After completing the survey, participants played roulette online to examine whether betting patterns adhered to the gambler’s fallacy. *Results:* Gambling involvement was significantly related to problem gambling severity for the community sample but not for the student sample. A logistic regression analysis that tested the involvement, motivation, impulsivity and cognitive correlates showed that money motivation and gambling related cognitions were the only significant independent predictors of gambling severity. Adherence to the gambler’s fallacy was stronger for students than for the community sample, and was associated with gambling related cognitions. *Discussion:* The motivational, impulsivity and cognitive, and correlates of problem gambling function similarly in university student gamblers and in gamblers from the general adult community. Interventions for both groups should focus on the financial and cognitive supports of problem gambling.

## Introduction

The widespread introduction of legalized gambling has spurred intensive study of the factors that place individuals at risk for the potential harms that gambling produces. A significant variable that has attracted recent attention is whether the gambler is a university student. University students provide an ideal population to study early stages of legalized gambling, and in particular the influence of their emerging personality dispositions on gambling severity. Studies of gambling in Canada, the United States and Great Britain show that approximately 75% of adults participate in gambling activities (Afifi, Cox, Martens, Sareen & Enns, 2010; Kessler et al., 2008; Wardle et al., 2011). Estimates of gambling participation by university students are inconsistent. Although LaBrie, Shaffer, LaPlante and Wechsler (2003) found that only 42% of university students reported gambling in the past year, Barnes, Welte, Hoffman and Tidwell (2010) reported a 75% rate of gambling participation by university students.

There is also disagreement on whether there is a difference in the prevalence of problem or pathological gambling among university students and in the general adult population. Barnes et al. (2010) found no difference between college students and non-college young adults in the frequency of gambling and the prevalence of problem gambling. However, college student status was associated with problem drinking. More recently, Gainsbury, Russell and Blaszczynski (2012) reported that the proportion of possible problem gamblers was lower among university students participating in research for course credit (8.8%) than in the general populations (17%), although there were no differences in gambling problems between students who participated for course credit and students who did not participate for course credit. It should be noted that whereas there were more females than males among the for-credit students, there were more males than females among the general population students recruited online. Most studies converge on the conclusion that the rate of problem gambling is higher in university students than in the general population (e.g., Blinn-Pike, Worthy & Jonkman, 2007). The anomalous finding reported by Gainsbury et al. (2012) may reflect their recruitment of the “general population” at online sites. It is not clear whether those gamblers are representative of the general adult gambling population.

Shaffer and Hall (2001) conducted a meta-analysis to compare the prevalence of different levels of gambling severity among North American college students and in the general population. Three levels of gambling severity were identified: level 1 referred to non-gamblers and gamblers who experienced no problems; level 2 referred to problem or at-risk gamblers who experienced problems at a sub-clinical level; and, level 3 referred to pathological gamblers. The meta-analysis yielded 85 estimates of the lifetime prevalence of the three levels of gambling severity. For adults in the general population the mean lifetime prevalence estimates for levels 1, 2, and 3 were 93.92%, 4.15%, and 1.92%, respectively. For college students the corresponding prevalence estimates were 83.13%, 10.88%, and 5.56%. These results suggest that, if problem gambling is defined as including both level 2 and level 3 gamblers, then the prevalence of problem gambling is at least twice as high among university students as among the general adult population.

Recent studies confirm the higher prevalence of disordered gambling among university students than in the general adult population. Across studies, the rate of problem gambling in the general adult population tends to fall between 1% and 5% (e.g., Jackson, Wynne, Dowling, Tomnay & Thomas, 2010; Kessler et al., 2008; MacLaren, Fugelsang, Harrigan & Dixon, 2011; Odlaug, Schreiber & Grant, 2013; Orford, Griffiths & Wardle, 2013). Barnes et al. (2010) found that 6% of college students were at-risk or problem gamblers. Moore et al. (2013) reported that the prevalence of moderate risk gamblers and problem gamblers among students in Australian universities was 8.5% and 5.4%, respectively. Nowak and Aloe (2013) carried out a meta-analysis of studies of gambling by university students in North America, Scotland, Nigeria, Singapore, Japan, and China. They reported that the prevalence of pathological gambling was 10.23%. The variation in estimates of the prevalence of levels of gambling severity across studies may be due to a host of variables including the measurement instruments, scoring thresholds, the time frame in which behaviors are described, and the method of survey administration (Williams, Volberg & Stevens, 2012).

In a recent direct comparison of university student gamblers and gamblers in the general population, Gainsbury et al. (2012) reported that university student gamblers were less likely to gamble on the Internet, had fewer gambling problems, had a more negative attitude toward gambling, and held more irrational beliefs. It is uncertain whether any of those differences reflect differences in recruitment methods: students participated in partial fulfillment of course requirements whereas community gamblers were recruited online. Although the groups differ in age, it is not known whether motivational, personality, and cognitive predictors of problem gambling severity function differently in university student gamblers and gamblers in the general adult population. The primary purpose of the present study was to begin to fill that gap. Group differences in the associations among the correlates and problem gambling severity would suggest that interventions be specialized for the different populations.

### Motivation and gambling

Several models of motivation for gambling have been proposed (e.g., Binde, 2013; Clarke et al., 2007; Fang & Mowen, 2009; Lee, Chae, Lee & Kim, 2007; McGrath, Stewart, Klein & Barrett, 2010; Neighbors, Lostutter, Cronce & Larimer, 2002; Thomas, Allen & Phillips, 2009; Wood & Griffiths, 2007). In a recent conceptual development, Binde (2013) identified four optional motives for gambling (the dream of hitting the jackpot; social rewards; intellectual challenge; mood change) and a core essential motive, the chance of winning. However, Binde (2013) did not provide any empirical tests of the model. Most other models assume that the main motivations for gambling include: excitement, money, avoidance (escape from problems), socialization, and, amusement. Lee et al. (2007) found that in a sample of university students the only motivational factor directly related to problem gambling severity was the monetary factor. Thomas et al. (2009) assessed the motivations of a community sample of electronic machine gamblers and found that escape was a significant motivator. Thomas et al. (2009) did not, however, measure the monetary motive. The motivation scale developed by Lee et al. (2007) was used in the present study. The scale measures five factors related to the motivations for gambling: excitement (e.g., “I enjoy the thrilling experience in risk”); money (e.g., “I want to win money easily”); avoidance (e.g., “I feel troubled”); socialization (e.g., “I meet new people”); and, amusement (e.g., “I enjoy leisure time”).

### Impulsivity and gambling

Theoretical advances in the measurement of impulsivity (e.g., Cyders, Smith, Spillane, Fischer, Annus & Peterson, 2007) underlie several recent studies of the dimensions that predict problem gambling (MacLaren et al. 2011; Michalczuk, Bowden-James, Verdejo-Garcia & Clark, 2011; Odlaug et al., 2013; Torres et al., 2013). Cyders et al. (2007) defined the components of impulsivity as follows: lack of planning involves a failure to plan ahead (e.g., disagreement on “I like to stop and think things over before I do them”); lack of perseverance involves a failure to maintain vigilant attention on a task (e.g., “There are so many little jobs that need to be done that I sometimes just ignore them all”); sensation seeking is the tendency to pursue novel or thrilling experiences (e.g., “I sometimes like doing things that are a bit frightening”); negative urgency is the tendency to act rashly when upset (e.g., “I often make matters worse because I act without thinking when I am upset”); and, positive urgency is the tendency to act rashly when experiencing an unusually positive mood (e.g., “When overjoyed, I feel like I can’t stop myself from going overboard”).

Michalczuk et al. (2011) found that the greatest differences between a clinical sample of pathological gamblers and healthy controls occurred on the negative and positive urgency components. The groups also differed significantly on lack of planning and lack of perseverance, but not on sensation seeking. In a meta-analysis of impulsivity studies that included several measures related to the Cyders et al. (2007) components (excluding positive urgency), MacLaren et al. (2011) concluded that only negative urgency and low planning differentiate problem gamblers and controls.

Torres et al. (2013) tested 21 pathological gamblers in rehabilitation, 20 cocaine-dependent individuals in rehabilitation, and 23 controls. Negative urgency and lack of planning were the only significant predictors of clinical status (i.e., in rehabilitation vs. control). No measures of impulsivity differentiated the cocaine and gambling groups. Severity of gambling was indexed for the gamblers by scoring interviews that asked about the number of months they had gambled and the amount of money spent on gambling per month. Only negative urgency was related to gambling severity. The distinct personality profiles of participants across studies may have contributed to whether positive urgency is a significant predictor of problem gambling (Michalczuk et al., 2011) or not (Torres et al., 2013).

### Cognition and gambling

Problem gambling is sustained by distorted gambling cognitions (Lund, 2011; Myrseth, Brunborg & Eidem, 2010). The Gambling Related Cognitions Scale (Raylu & Oie, 2004) identifies five cognitive factors related to gambling in a community sample: expectancies (e.g., “gambling makes the future brighter”); illusion of control (e.g., “specific numbers and colors can help increase my chances of winning”); predictive control (e.g., “losses when gambling are bound to be followed by a series of wins”); inability to stop (e.g., “I can’t function without gambling”); and, interpretive bias (e.g., “relating my losses to probability makes me continue gambling”). Emond and Marmurek (2010) found that those gambling related cognitions mediate the influence of irrational thinking styles on gambling severity.

Michalczuk et al. (2011) tested the relative contributions of gambling cognitions and impulsivity to gambling severity. They found that the effect sizes for differences between pathological gamblers seeking treatment and healthy community control participants were larger on gambling related cognitions than on impulsivity. The present study was designed to analyze the distinctive contributions of impulsivity and gambling related cognitions to gambling severity across a broader spectrum of gamblers in the general adult population and in university students.

### Gender and gambling

The literature on the relationship between gambling severity and gender has yielded mixed results (Afifi et al., 2010; Faregh & Leth-Steensen, 2011; Ko, Yen, Chen, Chen & Yen, 2005; LaPlante, Nelson, LaBrie & Shaffer, 2006; Wenzel & Dahl, 2009). However, there are consistent findings that the patterns of gambling differ for males and females. LaPlante et al. (2006) found that whereas males are more likely than females to prefer casino games, females are more likely than males to prefer slot machine games. Similarly, Faregh and Leth-Steensen (2011) identified eight classes of gamblers among which gender was a discriminating factor. For example, Class 2 gamblers who preferred non-strategic gambles such as lottery and scratch tickets comprised mainly females. In contrast, Class 4 gamblers were mostly males who preferred cards and games of skill. Michalczuk et al. (2011) did not carry out any analyses of gender differences given that their samples comprised 28 males in each group of 30 pathological gamblers and 30 healthy controls. The present study tested larger and more equally represented samples to explore gender-based differences in the impulsivity and cognitive correlates of gambling severity tested by Michalczuk et al. (2011).

### Gambling correlates and gambling behavior

The major goal of the present study was to compare university student gamblers and community gamblers on the relationship between problem gambling severity and the correlates of motivation, impulsivity, gambling related cognitions, and gender. A secondary aim was to assess whether the correlates predicted gambling behavior. The specific behavior selected was the betting choice while playing an online roulette game. It was expected that the primary predictor of gambling behavior would be cognitive (Michalczuk et al., 2011; Odlaug, Chamberlain, Kim, Schreiber & Grant, 2011).

Michalczuk et al. (2011) found that the tendency to prefer immediate small rewards to delayed larger rewards was stronger in pathological gamblers than in healthy controls. This discounting effect is typically interpreted as reflecting impulsivity. However, the discounting effect reported by Michalczuk et al. was more strongly associated with gambling related cognitions than with self-reported impulsivity. The discounting paradigm does not entail any direct risk of losses that are typical of gambling. Thus, the present study sought to determine whether gambling related cognitions would be a stronger determinant of gambling behavior during actual gambling than would impulsivity (and other gambling correlates).

Betting choices during a roulette game may disclose endorsement of the gambler’s fallacy. According to the gambler’s fallacy, a finite series of two equally probable events (e.g., heads and tails on a fair coin toss) should contain an equivalent number of instances of each event (e.g., as many heads as there are tails). Evidence that the gambler’s fallacy governs the betting of roulette players was reported by Croson and Sundali (2005) who analyzed the security videotapes of roulette bets provided by executives at a Reno Nevada casino.

Croson and Sundali (2005) found that gamblers were more likely to bet on a reversal in the color of the winning number as the number of prior successive instances of a winning color increased. For example, whereas the likelihood of betting on a color reversal was approximately 50% following a single instance of a color, that likelihood increased to 65% following a sequence of five instances of a color. Croson and Sundali did not have access to data on the gambling correlates that might predict the alternation betting pattern indicative of the gambler’s fallacy. The present study measured whether sample (students vs. community), gender, gambling motivation, gambling related cognitions, and impulsivity predict sensitivity to the recent history of outcomes when betting on the color of the winning number in a roulette game.

### Goals of the study

The present study measured associations among problem gambling severity, sample (students vs. community), gender, gambling motivation, impulsivity, and gambling related cognitions. LaPlante, Afifi and Shaffer (2013) found that among casino patrons gambling involvement, measured by the number of different types of games played, is a stronger predictor of gambling problems than is any specific type of game. The current study tested gambling involvement as a correlate of problem gambling severity with a broader spectrum of gamblers. Finally, the present study examined whether correlates of problem gambling severity are associated with the gambler’s fallacy as indexed by sensitivity to the recent history of outcomes when betting on the color of the winning number in a roulette game.

## Methods

### Participants

The student sample comprised 123 (64 males; 59 females) undergraduates no younger than 19 years, the legal age for gambling. Participants reported their age by selecting among six categories of age intervals where the lowest interval was 19–24 and all remaining intervals covered a 10-year span. In the university student sample, 98% of participants were in the youngest age category. Age was verified by a legal picture document such as a driver’s license. The students’ participation partially fulfilled a research credit component of their psychology course in which students selected from a wide range of research opportunities. It should be noted that the gender distribution in the undergraduate course is typically 66% female. The gender distribution in this study’s sample may more closely approximate the gender distribution of university student gamblers (Moore et al., 2013).

The 113 participants (51 males; 62 females) in the adult community gamblers sample were recruited by sampling from a database of gamblers who had participated previously in unrelated gambling studies. Only 15% of the community sample was in the youngest age range. The median age interval for the community sample was 45–54. The two samples differed significantly in their age distribution, *χ*^2^ (5, N = 236) = 172.63, *p* < .001.

Recruitment messages that were given by phone (to the community gamblers) or posted on the psychology research participant website stated that “The purpose of the study is to examine characteristics of gamblers”. All participants were paid $30 to cover their time and travel to the off-campus gambling laboratory.

### Measures

Participants completed a survey containing a series of standardized scales that measured problem gambling severity, gambling motivation, impulsivity, and gambling related cognitions. The survey also included questions about the forms of gambling the respondent had participated in during the last 12 months, their age, and their gender.

*Problem Gambling Severity Index* (PGSI). The PGSI (Ferris & Wynne, 2001) is a nine item subset of the Canadian Problem Gambling Inventory. Respondents are asked to think about the past year and to indicate the frequency for each item using a 4-point scale: 0 = never; 1 = sometimes; 2 = most of the time; 3 = almost always. The nine items are: How often have you bet more than you could really afford to lose? How often have you needed to gamble with larger amounts of money to get the same feeling of excitement? How often have you gone back another day to try to win back the money you lost? How often have you borrowed money or sold anything to get money to gamble? How often have you felt that you might have a problem with gambling? How often have people criticized your betting or told you that you had a gambling problem, regardless of whether or not you thought it was true? How often have you felt guilty about the way you gamble or what happens when you gamble? How often has your gambling caused you any health problems, including stress or anxiety? How often has your gambling caused any financial problems for you or your household?

The PGSI has been judged as superior to the Victorian Gambling Screen and the South Oaks Gambling Screen as a screen of problem gambling (McMillen & Wenzel, 2006). Holtgraves (2009) recommended the PGSI as an index of the progression of gambling severity in non-clinical samples. The PGSI total score across the nine items is used to classify respondents into gambling subtypes as follows: 0 = non-problem gambler; 1–2 = low risk gambler; 3–7 = moderate risk gambler; 8 or more = problem gambler. Ferris and Wynne (2001) described non-problem and low risk gamblers as not having experienced any adverse consequences from gambling. Moderate risk gamblers were described probabilistically (i.e., may or may not) in terms of having experienced adverse consequences from gambling. Problem gamblers were described as having experienced adverse consequences from gambling. In the present study, Cronbach’s alpha for the PGSI was 0.85.

*Gambling motivation.* The Lee et al. (2007) five-factor gambling motivation scale was adapted to a 27 item scale on which respondents indicated their level of agreement using a 5-point scale where 1 = strongly disagree, 3 = neutral, and 5 = strongly agree. The Cronbach’s alpha for the five separate factors in the present study were: amusement, .72; avoidance, 0.87; excitement, 0.90; monetary, 0.75; and, socialization, .81.

*Gambling Related Cognitions Scale (GRC)*. The GRC (Raylu & Oie, 2004) contains 23 items for which participants indicate their level of agreement with each statement on a 7-point scale (1 = strongly disagree; 7 = strongly agree). In the present study, Cronbach’s alpha for the total scale was 0.93. The alpha values for the subscales were as follows: expectancies, 0.82; illusion of control, 0.77; predictive control, 0.77; inability to stop, 0.86; interpretive bias, 0.84. *Impulsivity.* The Impulsivity scale (Cyders et al., 2007) contains 59 items for which respondents indicate their level of agreement using a 4-point scale from 1 = agree strongly to 4 = disagree strongly. The Cronbach’s alpha indices of internal consistency for the separate factors in the current study were: lack of deliberation, .87; lack of persistence, .85; sensation seeking, .91; positive urgency, .96; and, negative urgency, .90.

### Procedure

Participants were tested in groups of up to four participants. Each participant was tested in a separate cubicle in the off-campus gambling laboratory. After signing an informed consent document, participants completed a survey that included questions about gender, the types of gambling activities engaged in over the past year, and the standardized PGSI, GRC, Motivation, and Impulsivity scales. The survey was completed online or in paper and pencil format according to the preference of the participant.

In the final stage of the session, participants were asked to play a simulated American-style roulette game at www.rouletterus.com. The game was set in the free play mode and the player was given a $1000 stake. Participants were asked to play to a maximum of 15 minutes or until their funds were exhausted. Participants were told to try to maximize their winnings because the top 10 scorers would receive a $50 MasterCard gift card. Only “outside bets” on a single color were permitted and the maximum bet permitted on a spin was $100. The color and number of the winning numbers on the eight most recent spins was continuously updated on the screen as was the amount of money available to the player. Each player was observed by a separate research assistant who maintained a record of the color selected by the player and the color of the winning number on each bet.

### Ethics

Recruitment of participants followed approval of the study from our Research Ethics Board (REB). The REB application process requires providing the committee with copies of the recruitment posting, consent form, debriefing form, and a detailed description of the methods including materials. Upon arrival at the laboratory, participants completed the consent form which outlined the general purpose and method of the study. Participants were informed about their compensation, and that they were free to withdraw at any time without penalty. At the conclusion of their participation, participants received the debriefing form which included contacts for obtaining further information about the study and its findings.

## Results

Statistical analyses were carried out with SPSS-20 and, where necessary, are reported with corrections for violations of assumptions about variance. Alpha was set at .05.

### PGSI categories

The PGSI scores were subjected to a 2 × 2 Gender [male, female]) × Sample [student, community]) random groups analysis of variance (ANOVA). The mean overall PGSI score was 2.39 (*SD* = 3.28) and varied with neither Gender nor Sample (both *p* > .25). Table 1 presents the distribution of participants by gender across the four PGSI categories defined by Ferris and Wynne (2001). The distribution of participants across the gambling severity categories was: non-problem, 34%; low risk, 34%; moderate risk, 24%; and problem, 8%. The distribution of gambling severity categories was independent of gender, *χ*^2^ (3) = 2.57, *p* = .46, and was independent of sample, *χ*^2^ (3) = 0.54, *p* = .91.

**Table 1.** Distribution of participants by gender and sample across PGSI categories

		PGSI subtype			
		Non-problem	Low risk	Moderate risk	Problem
PGSI score		0	1–2	3–7	>7
Gender	Male	36	37	30	12
	Female	44	43	27	7
Sample	Students	40	41	31	11
	Community	40	39	26	8
	Total	80	80	57	19

The main analyses focused on a dichotomous comparison of non-problem and problem gamblers (cf. Afifi et al., 2010; Currie, Hodgins, Wang, El-Guebaly, Wynne & Chen, 2006; LaPlante et al., 2013; Orford et al., 2013; Williams & Wood, 2007). Participants with PGSI scores lower than 3 were categorized as “non-problem” gamblers, and those with PGSI scores above 2 were categorized as “problem” gamblers. The pooling of the non-problem and low-risk groups is consistent with the description of those categories as not having experienced adverse consequences of gambling (Ferris & Wynne, 2001). In the critical comparison study conducted by Michalczuk et al. (2011), the healthy controls scored a maximum of 2 on the PGSI which is consistent with the present pooling of the non-problem and low risk categories. In the present study, the mean PGSI scores for the non-problem and problem groups were 0.67 (*SD* = 0.75) and 6.04 (*SD* = 3.59), respectively, *t* (78) = 12.92, *p* < .001, Cohen’s *d* = 2.07. The groups differed on each of the nine PGSI questions with all *t*’s > 5, *p* < .001.

### Group differences in forms of gambling

The survey asked participants to indicate the forms of gambling in which they had participated during the previous year. The gamblers were also asked whether they had traveled outside the province to a slot machine or casino venue. The most popular forms of gambling were scratch cards (70%), lottery (62%), and slot machines (46%). Other forms of gambling listed were casinos (26%), card games (24%), horses (19%), bingo (17%), and Internet gambling (5%). The percentage of gamblers who traveled outside the province to play at a slot machine or casino venue was 16% and 11%, respectively. A 2 × 2 × 2 (PGSI Group [non-problem, problem] × Gender [male, female]) × Sample [student, community] ANOVA of the total number of gambling forms listed showed that all three main effects were statistically significant: PGSI group, *F*(1, 228) = 6.65, *p* = .011, η_p_^2^ = .03; Gender, *F*(1, 228) = 7.75, *p* = .006, η_p_^2^ = .03; and, Sample, *F*(1, 228) = 43.04, *p* < .001, η_p_^2^ = .16. There was also a significant PGSI group × Sample interaction, *F*(1, 228) = 11.28, *p* = .001, η_p_^2^ = .05.

The main effects indicated that problem gamblers played more types of games (*M* = 3.35, *SD* = 2.23) than did non-problem gamblers (*M* = 2.78, *SD* = 1.46), female gamblers (*M* = 3.37, *SD* = 1.96) played more types of games than did male gamblers (*M* = 2.76, *SD* = 1.46), and community gamblers played more types of games (*M* = 3.79, *SD* = 1.98) than did student gamblers (*M* = 2.33, *SD* = 1.28). The PGSI group × Sample interaction was probed by examining the PGSI effect for each sample. The student sample did not yield a significant difference in gambling involvement between problem gamblers (*M* = 2.21, *SD* = 1.24) and non-problem gamblers (*M* = 2.42, *SD* = 1.30), *F*(1, 122) < 1. In contrast, the problem gamblers in the community sample had a significantly higher rate of gambling involvement (*M* = 4.41, *SD* = 2.58) than did community non-problem gamblers (*M* = 3.13, *SD* = 1.53), *F*(1, 111) = 10.64, *p* = .001, η_p_^2^ = .09.

Further analyses of the gambling forms focused on the particular games. Table 2 displays the participation rates in each form of gambling by sample and PGSI group. A 2 × 2 × 2 (PGSI Group [non-problem, problem] × Gender [male, female]) × Sample (students, community) multivariate analysis of variance on the proportion of gamblers who engaged in each form of gambling showed all three main effects were statistically significant: PGSI group, Wilks’ Lambda = 0.92, *F*(10, 219) = 1.86, *p* = .05, η_p_^2^ = .08; Gender, Wilks’ Lambda = 0.90, *F*(10, 219) = 2.46, *p* = .01, η_p_^2^ = .10; and, Sample, Wilks’ Lambda = 0.67, *F*(10, 219) = 10.89, *p* < .001, η_p_^2^ = .33. The PGSI Group × Sample interaction was also significant, Wilks’ Lambda = 0.91, *F*(10, 219) = 2.15, *p* = .02, η_p_^2^ = .09. The upper portion of Table 3 shows that the problem gambling group was more likely than the non-problem group to participate in gambling involving cards, the Internet, slots outside the province, and casinos outside the province. The only form of gambling that significantly differentiated the genders was scratch cards. Females were more likely to report playing scratch cards (76%) than were males (63%), *χ*^2^ (1, *n* =236) = 4.42, *p* = .04. The lower portion of Table 3 shows that there were several significant differences among the gambling forms in which the two samples had participated. Students were more likely to purchase scratch cards than were the community adults. Community adults were more likely than students to engage in the other forms of gambling.

**Table 2.** Participation rates for the forms of gambling by sample and PGSI group (%)

	Students		Comminity	
Gambling form	Non-problem	Problem	Non-problem	Problem
	(*n* = 81)	(*n* = 42)	(*n* = 79)	(*n* = 34)
Scratch	71	79	58	68
Lottery	40	54	72	85
Slots	17	33	61	76
Casino	5	19	29	38
Cards	16	34	22	32
Horses	12	17	19	35
Bingo	16	17	18	15
Slots/travel	6	7	18	44
Casino/travel	4	7	13	29
Internet	1	2	5	18

**Table 3.** Effects of PGSI group and sample on participation rates for the forms of gambling (%)

	PGSI group		
	Non-problem	Problem	*χ*^2^ (1)
	(*n* = 160)	(*n* = 76)	
Cards	19	33	5.77^*^
Internet	3	9	3.95^*^
Slots/travel	12	24	5.44^*^
Casino/travel	8	17	4.99^*^
	Sample		
	Students	Community	*χ*^2^ (1)
	(*n* = 123)	(*n* = 113)	
Scratch	78	61	8.18^**^
Lottery	50	76	17.62^***^
Slots	28	65	33.98^***^
Casino	14	32	11.01^***^
Horses	14	24	3.94^*^
Slots/travel	7	26	16.35^***^
Casino/travel	5	18	9.88^**^
Internet	2	9	6.37^**^

*Note:* ^*^p < .05; ^**^p < .01; ^***^p < .001.

The PGSI Group × Sample interaction was tested by comparing the participation rates of non-problem and problem gamblers in each form of gambling separately for the two samples. For the student sample, the participation rate was higher for problem than for non-problem gamblers in the following types of gambling: slots, *χ*^2^ (1, *n* =123) = 4.05, *p* = .04; casino, *χ*^2^ (1, *n* =123) = 6.25, *p* = .01; and, cards, *χ*^2^ (1, *n* =123) = 4.82, *p* = .03. For the community sample, the participation rate was higher for problem than for non-problem gamblers in the following types of gambling: travel to slots, *χ*^2^ (1, *n* =113) = 7.70, *p* = .01; travel to casino, *χ*^2^ (1, *n* =113) = 4.58, *p* = .03; and, the Internet, *χ*^2^ (1, *n* =113) = 5.08, *p* = .02.

### Group differences in gambling motivation

The upper portion of Table 4 presents the means and standard deviations of the motivation scales. A 2 × 2 × 2 (PGSI Group [non-problem, problem] × Gender [male, female]) × Sample [student, community] multivariate ANOVA of the mean scores on the five motivation scales (Lee et al., 2007) showed that only PGSI Group emerged as a statistically significant main effect, Wilks’ Lambda = 0.77, *F*(5, 224) = 13.32, *p* < .001, η_p_^2^ = .23. There were no significant interactions. The upper portion of Table 5 summarizes the analyses of the PGSI Group differences on the motivation scales. Problem gamblers scored significantly higher than non-problem gamblers on amusement, avoidance, and money. As in Lee et al. (2007), the largest effect size was for monetary motivation.

### Group differences in Gambling Related Cognitions (GRC)

The means and standard deviations on the gambling related cognition scales are presented in the middle portion of Table 4. A 2 × 2 × 2 (PGSI Group [non-problem, problem] × Gender [male, female]) × Sample (students, community) multivariate ANOVA of the means on the subscales of the GRC showed that the only statistically significant effects were for the main effects of PGSI Group, Wilks’ Lambda = 0.72, *F*(5, 224) = 17.56, *p* < .001, η_p_^2^ = .28, and Gender, Wilks’ Lambda = 0.95, *F*(5, 224) = 2.36, *p* = .04, η_p_^2^ = .05. The middle portion of Table 5 shows that the PGSI Group effect was significant on all the GRC subscales. These results replicate the finding that the problem gamblers have higher scores on the GRC scales than do non-problem gamblers (Emond & Marmurek, 2010; Michalczuk et al., 2011). The only scale on which Gender had a significant effect was interpretive bias. Males scored higher on interpretive bias (*M* = 3.09, *SD* = 1.49) than did females (*M* = 2.44, *SD* = 1.34), *F*(1, 228) = 7.15, *p* = .008, *d* = 0.46.

**Table 4.** Means and standard deviations on gambling motivation, gambling cognition, and impulsivity

	Students				Community			
Scale	Non-problem (*n* = 81)		Problem (*n* = 42)		Non-problem (*n* = 79)		Problem (*n* = 34)	
	*M*	*SD*	*M*	*SD*	*M*	*SD*	*M*	*SD*
Motivation								
Amusement	3.29	0.89	4.00	0.55	3.35	0.81	3.36	0.74
Avoidance	1.89	0.76	2.44	0.79	1.66	0.65	2.25	0.81
Excitement	3.46	0.93	3.89	0.64	3.54	0.81	3.56	0.74
Money	2.89	.90	3.76	0.66	2.76	0.87	3.52	0.61
Social	3.25	0.91	3.74	0.78	3.35	0.89	3.29	0.98
Gambling-related cognition								
Inability to stop	1.27	0.62	2.26	1.34	1.27	0.54	2.89	1.19
Interpretive bias	2.43	1.38	3.81	1.29	2.27	1.24	3.35	1.45
Expectancies	2.36	1.37	3.68	1.37	2.27	1.03	3.53	1.20
Illusion of control	1.81	1.21	2.61	1.28	1.60	0.73	2.32	1.18
Predictive control	2.22	1.14	3.28	1.10	2.17	0.96	2.89	1.06
Impulsivity								
Negative urgency	2.29	0.52	2.58	0.47	2.22	0.58	2.46	0.55
Planning (lack of)	2.87	0.49	2.77	0.33	2.86	0.51	2.95	0.33
Perseverance	2.65	0.59	2.69	0.24	2.71	0.54	2.69	0.24
Positive urgency	1.89	0.58	2.30	0.54	1.88	0.54	2.06	0.57
Sensation-seeking	2.81	0.73	2.85	0.55	2.69	0.66	2.52	0.49

**Table 5.** Effects of PGSI subtype on gambling motivation, gambling cognitions, and impulsivity

	PGSI subtype						
Scale	Non-problem		Problem		*F*(1, 228)	*p*	Cohen’s *d*
	*M*	*SD*	*M*	*SD*			
Motivation							
Amusement	3.32	0.85	3.83	0.67	18.86	^***^	0.66
Avoidance	1.78	0.71	2.35	0.80	30.53	^***^	0.75
Excitement	3.49	0.87	3.74	0.70	3.16	.08	0.32
Money	2.83	0.88	3.65	0.67	48.11	^***^	1.05
Social	3.30	0.90	3.54	0.93	2.49	.12	0.26
Gambling-related cognition							
Inability to stop	1.27	0.58	2.27	1.27	65.16	^***^	1.01
Interpretive bias	2.35	1.31	3.60	1.37	41.88	^***^	0.93
Expectancies	2.32	1.10	3.62	1.29	59.71	^***^	1.08
Illusion of control	1.70	1.01	2.32	1.18	25.11	^***^	0.56
Predictive control	2.19	1.05	3.11	1.09	34.74	^***^	0.86
Impulsivity							
Negative urgency	2.25	0.55	2.53	0.51	13.41	^***^	0.53
Perseverance	2.68	0.56	2.60	0.41	0.02	.89	0.02
Planning (lack of)	2.87	0.50	2.85	0.34	0.01	.81	0.05
Positive urgency	1.88	0.56	2.19	0.57	15.53	^***^	0.55
Sensation-seeking	2.75	0.70	2.70	0.55	0.59	.44	0.08

*Note:* ^***^p < .001.

### Group differences in impulsivity

The means and standard deviations on the Impulsivity scales (Cyders et al., 2007) are presented in lower portion of Table 4. A 2 × 2 × 2 (PGSI Group [non-problem, problem] × Gender [male, female]) × Sample (students, community) multivariate analysis of variance of the means on the Impulsivity scales showed that the only significant effect was for PGSI Group, Wilks’ Lambda = 0.92, *F* (5, 224) = 3.73, *p* = .003, η_p_^2^ = .08. The lower portion of Table 5 shows that the PGSI Groups differed significantly on only the positive urgency and negative urgency scales. These effects replicate the findings of Michalczuk et al. (2011) that the two urgency scales yield the largest difference between problem and non-problem gamblers.

### Logistic regression of gambling correlates on PGSI classification

A logistic regression was run to test the relative strengths of the correlates at predicting membership in the two gambling severity groups. The multivariate analyses showed that non-problem and problem gamblers differed on three motivations for gambling: amusement; avoidance, and money. Those variables were entered separately into the logistic regression. The PGSI groups differed on all the gambling cognition sub-scales which were all significantly inter-correlated (mean *r* = .60). Accordingly, as in Michalczuk et al. (2011), the overall mean gambling cognition score was used a measure of the cognitive correlate of gambling severity. Positive urgency and negative urgency were the only impulsivity sub-scales that significantly differentiated the two gambling severity groups in the multivariate analysis. Those scales were entered separately into the logistic regression. The number of types of games played was also entered as the involvement correlate of gambling severity.

Table 6 summarizes the results of the logistic regression analysis. The Wald criterion is a *χ*^2^ (1) statistic used to test the coefficient B; Exp (B) is an Odds Ratio measure; the Hosmer and Lemeshow *χ*^2^ test provides a measure of goodness of model fit (a good fit is indexed by *p* > .05); and, the Nagelkerke R^2^ indexes explained variance. Multicollinearity was not a problem as indicated by the tolerance values. Gambling-related cognitions and monetary motivation were the only correlates that significantly predicted PGSI group. Avoidance motivation and gambling involvement (number of types of games played) were marginally significant predictors. Separate logistic regression analyses were run on the two samples and on the two genders. Each of those four analyses resulted in patterns similar to those in the overall analysis in that monetary motivation and cognition were significant predictors of PGSI group while impulsivity was not. However, gambling involvement was a marginally significant correlate for the both the community sample (Wald = 2.97, *p* = .085, Exp (B) = 1.26) and for females (Wald = 2.64, *p* = .10, Exp (B) = 1.24), but was not significant for the student sample (Wald = 0.32, *p* = .57) and males (Wald = 1.55, *p* = .21).

**Table 6.** Summary of logistic regression on PGSI category

Variable	Wald	Exp (B)	Exp (B) [95% C]I	Tolerance
Cognition	11.97^***^	2.19	[1.41, 3.42]	.59
Money	11.39^***^	2.51	[1.47, 4.29]	.57
Avoidance	2.76^*^	1.53	[0.93, 2.54]	.70
Amusement	0.49	1.24	[0.67, 2.30]	.60
Positive urgency	0.62	1.38	[0.62, 3.08]	.57
Negative urgency	0.01	1.00	[0.48, 2.30]	.54
Involvement	2.98^*^	1.17	[0.98, 1.40]	.94

Nagelkerke R^2^ = .42; Hosmer and Lemeshow *χ*^2^ (8) = 8.56; *p* = .38; ^*^ *p* = .10; ^***^ *p* < .001.

### Analyses of roulette betting on color alternation

The top row of Table 7 displays the means and standard deviations for the number of occurrences of a run of consecutive outcomes of a given color during the roulette game for run lengths 1, 2, and 3. The median number of occurrences of longer runs was very low (2 for a run of 4; 1 for a run of 5; and, 0 for a run of 6 or longer). A one-way repeated measures ANOVA for run lengths 1 to 6 showed a statistically significant inverse relationship between run length and the likelihood of experiencing that run length during the roulette game, *F*(5, 280) = 833.66, *p* < .001, η_p_^2^ = .77. Because inclusion of the low frequency run lengths of 4, 5 and 6 in analyses of gambler group effects would compromise power due to the loss of participants, a separate one-way repeated measures ANOVA for run lengths 1, 2 and 3 was conducted to confirm the inverse relationship between run length and frequency of occurrence, *F*(2, 265) = 787.08, *p* < .001, η_p_^2^ = .76. Both the linear and quadratic trends were statistically significant: linear trend, *F*(1, 243) = 828.56, *p* < .001, η_p_^2^ = .77; quadratic trend, *F*(1, 243) = 373.20, *p* < .001, η_p_^2^ = .61.

**Table 7.** Mean run length frequency and probability of betting on a subsequent alternation

	Run length					
	1		2		3	
	*M*	*SD*	*M*	*SD*	*M*	*SD*
Frequency of outcome	24.83	12.69	11.45	5.93	5.28	3.18
Probability of alternation						
Students	.46	.12	.56	.24	.63	.26
Community	.49	.13	.52	.22	.58	.28
Overall	.47	.12	.54	.24	.61	.27

The lower portion of Table 7 presents the mean probability of betting on an alternation in the color of the winning number following a run of a given color. A 2 × 2 × 2 × 3 (PGSI Group [non-problem, problem] × Gender [male, female] × Sample (students, community) × Run [1, 2, 3]) mixed ANOVA with Run as a repeated measures factor showed that the likelihood of betting on an alternation in color increased as the run length of the previous color increased, *F*(2, 364) = 22.31, *p* < .001, η_p_^2^ = .10. The linear trend was statistically significant, *F*(1, 193) = 36.06, *p* < .001, η_p_^2^ = .16, but the quadratic trend was not, *F* (1, 210) < 1. There was a marginally significant Sample × Run interaction, *F*(2, 386) = 2.59, *p* = .08, η_p_^2^ = .01. Separate single factor repeated measures analyses showed that the effect of Run was larger for the students than for the community sample: students, *F*(2, 226) = 27.96, *p* < .001, η_p_^2^ = .19; community, *F*(2, 161) = 6.09, *p* = .004, η_p_^2^ = .06. There was also a marginally significant effect of Gender, *F*(1, 193) = 3.23, *p* = .07, η_p_^2^ = .02. Males were more likely to bet on a color alternation ((*M* = .56, *SD* = .16) than were females (*M* = .52, *SD* = .17).

Comparisons of the overall observed probability of a color alternation bet to an expected probability of .50 showed that the difference was significant only for a run length of 3, *z* = 3.20, *p* = .001. Gambling related cognitions, monetary motivation, avoidance motivation and gambling involvement (number of types of games played), the significant predictors of problem gambling severity in the binary logistic regression, were entered as simultaneous predictors of betting on an alternation following three successive outcomes of a single color. The regression model was statistically significant, *F*(2, 219) = 3.62, *p* = .007, adjusted R^2^ = .05. The only significant predictor, however, was the cognitive correlate, Beta = .24, *t* = 3.02, *p* = .002. All other *t*-values were < 1.5.

## Limitations

As noted by Gainsbury et al. (2012) and Williams et al. (2012), a critical restriction on establishing general principles from studies of gambling is the dependency on the methods of recruiting the sample. In the present study, all participants had engaged in at least one form of gambling during the past year. The university student gamblers received course credit and gamblers from the general community were experienced participants in unrelated gambling studies. In addition, all participants were compensated with a financial payment. Although the participants did not risk their own money while gambling, they were motivated during the roulette game by the prospect of winning an additional financial prize. It is uncertain whether any of those considerations limit the generality of the pattern of results beyond the problem and non-problem gamblers who participated in the study.

Another potential limitation in interpreting the gambling behavior results is that participants may have varied in their experience with roulette. Moreover, participants were allowed a maximum of 15 minutes of play during the roulette game. Although that time frame may be at variance with what players might select in a session within a true gambling environment, significant effects have been found in prior studies using a short period of time (e.g., Monaghan & Blaszczynski, 2010). Furthermore, although many important features of gambling environments were not represented in the laboratory (e.g., unlimited play time, a wider range of betting amounts, opportunities for diversions such as refreshments and entertainment), evidence of adherence to the gambler’s fallacy showed that it is possible to capture essential characteristics of gambling in a controlled laboratory environment. Perhaps the most convincing evidence that the laboratory evoked representative gambling behavior is that every participant elected to participate in the roulette game although they had the option to take $30 and leave.

## Discussion

Although the distribution of non-problem and problem gamblers was similar for university student gamblers and the community gamblers, the gambling behaviors of those groups differed in several respects. Community gamblers engaged in more different types of gambling. Involvement was higher for problem than for non-problem gamblers in the community sample but not in the student sample. The relationship between problem gambling and involvement for the community sample is consistent with the results of LaPlante et al. (2013) for casino patrons. The involvement difference between students and community adults was supported by the analysis of specific forms of gambling. Community adults were more likely than university students to engage in most forms of gambling including Internet gambling (cf. Gainsbury et al., 2012) and playing slots outside the province. Those two forms of gambling are associated with problem gambling severity. It may be that larger amounts of disposable income support the wider range of gambling activities accessible to the community adults.

The relationship between Internet gambling and problem gambling has been the focus of recent research. Griffiths, Wardle, Orford, Sproston and Erens (2009) reported a 6% prevalence rate of Internet gambling in a community survey and a 5% rate of problem gambling as defined by DSM-IV criteria among Internet gamblers. In a study designed to examine whether university student gamblers are representative of gamblers in the general community, Gainsbury et al. (2012) found that students whose participation fulfilled a course requirement were less likely to gamble on the Internet than were participants recruited on the Internet. Moreover, the sample recruited on the Internet had a higher proportion of problem gamblers (17%) than did the university student sample (9%). Gainsbury et al. (2012) defined problem gamblers as those scoring above 8 on the PGSI. The association between gambling on the Internet and problem gambling severity was replicated in the current study. Estimates of the rates of Internet participation and problem gambling differ across studies and are particularly high in Gainsbury et al. (2012). Their high rates of Internet play and problem gamblers suggest that recruitment over the Internet may inflate estimates of Internet gambling in the general population.

There were few differences between the student and community samples on gambling motivation and personality. For the student sample, males scored higher than females on excitement motivation. There was no gender effect for excitement motivation in the community sample. Students overall scored higher on sensation seeking than did the community sample. There were no sample effects on gambling related cognitions, although the student sample exhibited a stronger tendency to adhere to the gambler’s fallacy when betting on the color of the winning number in a roulette game. None of the motivation and personality measures on which the samples differed were significant correlates of gambling severity. Moreover, the motivation and personality measures that were significant correlates of gambling severity did not yield sample differences.

Problem gambling severity was associated with gambling motivation, impulsivity, and gambling related cognitions. Money was the strongest motivator. Amusement and avoidance of personal problems were also significant motivators. Whereas all the components of gambling related cognitions were related to problem gambling severity, the only significant impulsivity predictors of gambling severity were positive urgency and negative urgency. Gambling related cognitions and money motivation were the only significant predictors of gambling severity when all the correlates were entered into a logistic regression analysis.

The logistic regression analysis supports research showing that the impact of impulsivity on gambling severity is secondary to cognitive contributions. Michalczuk et al. (2011), in a comparison of pathological gamblers and healthy controls, reported a mean effect size (Cohen’s *d*) of 1.15 for the significant impulsivity factors and a mean effect size of 1.45 for the significant gambling related cognitions. In the present study, the differences between non-problem and problem gamblers yielded a mean effect size of 0.47 for the significant impulsivity factors (positive urgency and negative urgency) and a mean effect size of 0.89 for gambling related cognitions. The smaller effect sizes in the present study likely reflect the use of sub-clinical samples in contrast to Michalczuk et al. (2011) who compared pathological gamblers seeking treatment with healthy controls.

Chiu and Storm (2010) ran a multiple regression with 23 personality predictors of problem gambling in college students. The strongest predictor was impulsive nonconformity (Mason, Claridge & Jackson, 1995). However, gambling specific motivational and cognitive factors were not tested in that analysis so it is not known whether impulsive nonconformity effects are independent of cognitive and motivation correlates. If all generic measures of impulsivity emerge as indirect predictors of gambling severity, then it may be useful to develop gambling specific measures of impulsivity. Failures of emotional regulation while gambling, corresponding to positive urgency and negative urgency, are likely to be significant independent correlates of gambling severity (Torres et al., 2013).

Michalczuk et al. (2011) found that the preference for immediate small rewards over delayed larger rewards in a delay discounting paradigm was more strongly associated with gambling related cognitions than with self-reported impulsivity. The discounting paradigm presents choices between hypothetical rewards that have no financial impact on the research participants. In the present study, the participants played a roulette game in which the outcome held financial implications in that there was a prospect of winning a $50 gift card. The probability of betting on the color of a winning number increased with the number of consecutive preceding outcomes of the other color. Gambling cognitions, but neither motivation nor impulsivity, predicted this adherence to the gambler’s fallacy.

Hahn and Warren (2009) have argued that the “statistical properties of the experienced environment or … reasonable inferences from that experience” (p. 459) support adherence to the gambler’s fallacy. For example, participants in the present study witnessed a run length of 1 approximately five times as often as a run length of 3. The inference participants would make is that long run lengths are unlikely. The likelihood of betting according to that inference was predicted by the gambling related cognitions held by the participants.

Interventions designed to minimize harms associated with problem gambling should address the motivations and the cognitions that support problem gambling. The motivation to win money may be linked to strategic betting. Interventions may benefit from the inclusion of simulations of gambling such as roulette playing. Those simulations would serve to enhance awareness of the random pattern of outcomes and in turn reduce expectations that the likelihood of winning is enhanced by strategic betting. Reducing biases in games such as roulette where the odds of a specific outcome are well defined may generalize to games in which the odds of an outcome are generally unknown (e.g., slot machines).

Although motivation and cognitive correlates were the strongest independent predictors of problem gambling severity, there is consistent evidence that the impulsivity components of positive urgency and negative urgency differentiate problem and non-problem gamblers. It should be noted that the gambling related cognition scales include items that refer to emotional states (e.g., “gambling makes me happier” and “having a gamble helps reduce tension and stress” on the Expectancies sub-scale) and loss of control (e.g., “it is difficult to stop gambling as I am so out of control” and “I’m not strong enough to stop gambling” on the Inability to Stop sub-scale). Gambling specific measures that dissociate “cold” (non-emotional) and “hot” (emotional) supports of problem gambling (Torres et al., 2013) may promote the development of interventions tailored to the diversity of the mechanisms that support problem gambling. Such interventions would afford similar benefits to university student gamblers and adult community gamblers given the comparable associations among the correlates of gambling severity in those populations.

## References

[B1] Afifi T. O., Cox B. J., Martens P. J., Sareen J., Enns M. W. (2010). Demographic and social variables associated with problem gambling among men and women in Canada. Psychiatry Research.

[B2] Barnes G. M., Welte J. W., Hoffman J. H., Tidwell M. C. (2010). Comparisons of gambling and alcohol use among college students and noncollege young people in the United States. Journal of American College Health.

[B3] Binde P. (2013). Why people gamble; A model with five motivational dimensions. International Gambling Studies.

[B4] Blinn-Pike, L., Worthy S. L., Jonkman J. N. (2007). Disordered gambling among college students: a meta-analytic synthesis. Journal of Gambling Studies.

[B5] Chiu J., Storm L. (2010). Personality, perceived luck and gambling attitudes as predictors of gambling involvement. Journal of Gambling Studies.

[B6] Clarke D., Tse S., Abbott M. W., Townsend S., Kingi P., Manaia W. (2007). Reasons for starting and continuing gambling in a mixed ethnic community sample of pathological and non-problem gamblers. International Gambling Studies.

[B7] Croson R., Sundali J. (2005). The gambler’s fallacy and the hot hand: Empirical data from casinos. The Journal of Risk and Uncertainty.

[B8] Currie S. R., Hodgins D. C., Wang J., El-Guebaly N., Wynne H., Chen S. (2006). Risk of harm among gamblers in the general population as a function of level of participation in gambling activities. Addiction.

[B9] Cyders M. A., Smith G. T., Spillane N. S., Fischer S., Annus A. M., Peterson C. (2007). Integration of impulsivity and positive mood to predict risky behavior: Development and validation of a measure of positive urgency. Psychological Assessment.

[B10] Emond M. S., Marmurek H. H. C. (2010). Gambling related cognitions mediate the association between thinking style and problem gambling severity. Journal of Gambling Studies.

[B11] Fang X., Mowen J. C. (2009). Examining the trait and functional motive antecedents of four gambling activities: Slot machines, skilled card games, sports betting, and promotional games. Journal of Consumer Marketing.

[B12] Faregh N., Leth-Steensen C. (2011). The gambling profiles of Canadians young and old: Game preferences and play frequencies. International Gambling Studies.

[B13] Ferris J., Wynne H. (2001). The Canadian problem gambling index: Final report.

[B14] Gainsbury S. M., Russell A., Blaszczynski A. (2012). Are psychology student gamblers representative of non-university students and general gamblers? A comparative analysis. Journal of Gambling Studies.

[B15] Griffiths M., Wardle H., Orford J., Sproston K., Erens B. (2009). Sociodemographic correlates of Internet gambling: Findings from the 2007 British Gambling Prevalence Survey. Cyberpsychology & Behavior.

[B16] Hahn U., Warren P. A. (2009). Perceptions of randomness: Why three heads are better than four. Psychological Review.

[B17] Holtgraves T. (2009). Evaluating the Problem Gambling Severity Index. Journal of Gambling Studies.

[B18] Jackson A. C., Wynne H., Dowling N. A., Tomnay J. E., Thomas S. A. (2010). Using the CPGI to determine problem gambling prevalence in Australia: Measurement issues. International Journal of Mental Health and Addiction.

[B19] Kessler R. C., Hwang I., LaBrie R., Petukhova M., Sampson N. A., Winters K. C., Shaffer H. J. (2008). The prevalence and correlates of DSM-IV pathological gambling in the National Comorbidity Survey Replication. Psychological Medicine.

[B20] Ko C., Yen J., Chen C., Chen S., Yen C. (2005). Gender differences and related factors affecting online gaming addiction among Taiwanese adolescents. Journal of Nervous & Mental Disease.

[B21] LaBrie R. A., Shaffer H. J., LaPlante D. A., Wechsler H. (2003). Correlates of college student gambling in the United States. Journal of American College Health.

[B22] LaPlante D. A., Afifi T. O., Shaffer H. J. (2013). Games and gambling involvement among casino patrons. Journal of Gambling Studies.

[B23] LaPlante D. A., Nelson S. E., LaBrie R. A., Shaffer H. J. (2006). Men and women playing games: Gender and the gambling preferences of Iowa Gambling Treatment program participants. Journal of Gambling Studies.

[B24] Lee H.-P., Chae P. K., Lee H.-S., Kim Y.-K. (2007). The five-factor gambling motivation model. Psychiatry Research.

[B25] Lund I. (2011). Irrational beliefs revisited: Exploring the role of gambling preferences in the development of misconceptions in gamblers. Addiction Research and Theory.

[B26] MacLaren V. V., Fugelsang J. A., Harrigan K. A., Dixon M. J. (2011). The personality of pathological gamblers: A meta-analysis. Clinical Psychology Review.

[B27] Mason O., Claridge G., Jackson M. (1995). New scales for the assessment of schizotypy. Personality and Individual Differences.

[B28] McGrath D. S., Stewart S. H., Klein R. M., Barrett S. P. (2010). Self-generated motives for gambling in two population-based samples of gamblers. International Gambling Studies.

[B29] McMillen J., Wenzel M. (2006). Measuring problem gambling: Assessment of three prevalence screens. International Gambling Studies.

[B30] Michalczuk R., Bowden-Jones H., Verdejo-Garcia A., Clark L. (2011). Impulsivity and cognitive distortions in pathological gamblers attending the UK National Problem Gambling Clinic: A preliminary report. Psychological Medicine.

[B31] Monaghan S., Blaszczynski A. (2010). Impact of mode of display and message content of responsible gambling signs for electronic gaming machines on regular gamblers. Journal of Gambling Studies.

[B32] Moore S. M., Thomas A. C., Kale S., Spence M., Zlatevska N., Staiger P. K., Graffam J., Kyrios M. (2013). Problem gambling among international and domestic university students in Australia: Who is at risk?. Journal of Gambling Studies.

[B33] Myrseth H., Brunborg G., Eidem M. (2010). Differences in cognitive distortions between pathological and non-pathological gamblers with preferences for chance or skill games. Journal of Gambling Studies.

[B34] Neighbors C., Lostutter T. W., Cronce J. M., Larimer M. E. (2002). Exploring college student gambling motivation. Journal of Gambling Studies.

[B35] Nowak D. E., Aloe A. M. (2013). The prevalence of pathological gambling among college students; A meta-analytic synthesis, 2005–2013. Journal of Gambling Studies.

[B36] Odlaug B. L., Chamberlain S. R., Kim S. W., Schreiber L. R. N., Grant J. E. (2011). A neurocognitive comparison of cognitive flexibility and response inhibition in gamblers with varying degrees of clinical severity. Psychological Medicine.

[B37] Odlaug B. L., Schreiber L. R. N., Grant J. E. (2013). Personality dimensions and disorders in pathological gambling. Current Opinion in Psychiatry.

[B38] Orford J. F., Griffiths M. D., Wardle H. (2013). What proportion of gambling is problem gambling? Estimates from the 2010 British Gambling Prevalence Survey. International Gambling Studies.

[B39] Raylu N., Oie T. P. S. (2004). The Gambling-Related Cognition Scale (GRCS): Development, confirmatory factor validation and psychometric properties. Addiction.

[B40] Shaffer H., Hall M. (2001). Updating and refining meta-analytic prevalence estimates of disordered gambling behavior in the United States and Canada. Canadian Journal of Public Health.

[B41] Thomas A. C., Allen F. C., Phillips J. (2009). Electronic gaming machine gambling: Measuring motivation. Journal of Gambling Studies.

[B42] Torres A., Catena A., Megías A, Maldonado A., Cándido A., Verdejo-García A., Perales J. C. (2013). Emotional and non-emotional pathways to impulsive behavior and addiction. Frontiers in Human Neuroscience.

[B43] Wardle H., Moody A., Spence S., Orford J., Volberg R., Jotangia D., Griffiths M., Hussey D., Dobbie F. (2011). British gambling prevalence survey 2010.

[B44] Wenzel H. G., Dahl A. A. (2009). Female pathological gamblers – A critical review of the clinical findings. International Journal of Mental Health and Addiction.

[B45] Williams R. J., Volberg R. A., Stevens R. M. G. (2012). The population prevalence of problem gambling: Methodological influences, standardized rates, jurisdictional differences, and worldwide trends. Report prepared for the Ontario Problem Gambling Research Centre and the Ontario Ministry of Health and Long Term Care.

[B46] Williams R. J., Wood R. T. A. (2007). The proportion of Ontario gambling revenue derived from problem gamblers. Canadian Public Policy.

[B47] Wood R. T. A., Griffiths M. D. (2007). A qualitative investigation of problem gambling as an escape-based coping strategy. Psychology and Psychotherapy: Theory, Research and Practice.

